# Luminescent Ionogels with Excellent Transparency, High Mechanical Strength, and High Conductivity

**DOI:** 10.3390/nano10122521

**Published:** 2020-12-15

**Authors:** Lumi Tao, Yuchuan Liu, Dan Wu, Qiao-Hua Wei, Andreas Taubert, Zailai Xie

**Affiliations:** 1 Fujian Provincial Key Laboratory of Electrochemical Energy Storage Materials, College of Chemistry, Fuzhou University, 2 Xueyuan Road, Fuzhou 350002, China; taolumi2020@163.com (L.T.); liuyuchuan86@gmail.com (Y.L.); wdtazjs@163.com (D.W.); qhw76@fzu.edu.cn (Q.-H.W.); 2 Institute of Chemistry, University of Potsdam, 14476 Potsdam, Germany

**Keywords:** ionic liquid, ionogel, carbon dots, organic–inorganic hybrid, luminescence, mechanical strength

## Abstract

The paper describes a new kind of ionogel with both good mechanical strength and high conductivity synthesized by confining the ionic liquid (IL) 1-butyl-3-methylimidazolium bis(trifluoromethane sulfonyl)imide ([Bmim][NTf_2_]) within an organic–inorganic hybrid host. The organic–inorganic host network was synthesized by the reaction of methyltrimethoxysilane (MTMS), tetraethoxysilane (TEOS), and methyl methacrylate (MMA) in the presence of a coupling agent, offering the good mechanical strength and rapid shape recovery of the final products. The silane coupling agent 3-methacryloxypropyltrimethoxysilane (KH-570) plays an important role in improving the mechanical strength of the inorganic–organic hybrid, because it covalently connected the organic component MMA and the inorganic component SiO_2_. Both the thermal stability and mechanical strength of the ionogel significantly increased by the addition of IL. The immobilization of [Bmim][NTf_2_] within the ionogel provided the final ionogel with an ionic conductivity as high as ca. 0.04 S cm^−1^ at 50 °C. Moreover, the hybrid ionogel can be modified with organosilica-modified carbon dots within the network to yield a transparent and flexible ionogel with strong excitation-dependent emission between 400 and 800 nm. The approach is, therefore, a blueprint for the construction of next-generation multifunctional ionogels.

## 1. Introduction

Ionic liquids (ILs) have demonstrated great potential for applications in catalysis [1,2,3], separation [4,5], as electrolyte [6], and in sensors [7]. This is due to their extraordinary properties such as high chemical and thermal stability, high ion conductivity, affinity to inorganic compounds, and wide electrochemical stability windows. One key advantage of ILs is that the IL cations and anions can be adapted to provide an IL with specific properties and hence functionalities [8,9,10,11]. Immobilization of free ILs in a solid matrix generates macroscopic solids commonly termed ionogels (IGs). IGs combine the properties of the IL and the host matrix [12,13] and have been put forward for the development of macroscopic solid materials where such a combination of IL properties [14,15] (e.g., ionic conductivity [16] or thermal stability) and properties of a host (e.g., transparency [17,18] or mechanical stability [19]) provides new fields of application. As of now, IGs have been considered for use as solid electrolytes [20,21], solid actuators [22,23], and heterogeneous catalysts [24,25]. These applications often rely on good mechanical strength while maintaining high chemical stability and ionic conductivity. Therefore, new multifunctional IGs combining the above features are highly sought after [26,27].

Chemical and structural design of a solid matrix is crucial to tune the physical properties and chemical functions of IGs [14]. To date, numerous solid scaffolds have been used for IG fabrication. Typical examples include inorganic oxides and organic polymers, especially silica and poly(methyl methacrylate) (PMMA) [28,29,30]. With solvent-casting, a series of soft, magnetic, and luminescent IGs can be fabricated using PMMA as a host [31]. Here, ILs often lead to plasticization that reduces the glass transition temperature of IGs [32]. Moreover, polymer-based IGs often suffer from structural degradation, depolymerization, or leaching when exposed to polar solvents. This severely reduces their use in applications where there is a direct contact with liquid phases.

In contrast, IGs derived from inorganic oxides have better chemical stability [33] and higher ionic conductivity [34] when compared with polymer-based IGs. For example, silica-based IGs can exhibit a tunable phase behavior of ILs [35] and have ionic conductivities as high as 10^−4^ S cm^−1^. However, these IGs are brittle and only have a low mechanical strength. This currently prevents their application in the nascent field of flexible matters [36].

To overcome the issues of both polymer- and inorganic-based IGs, it has recently been proposed that the addition of inorganic nanoparticles to polymer-based IGs may improve the mechanical properties of the material [37,38,39]. However, the quality of an IG made by this method strongly depends on the properties of inorganic components [40] such as particle size. Moreover, the properties are further affected by processes happening during the IG synthesis, for example phase separation or particle aggregation [31]. 

As a result, there is a tremendous need for a chemically, electrochemically, and thermally robust IG host material combining the advantages of polymeric and inorganic host materials to afford a true next generation of IGs for application in modern technology. The current article addresses this question. The new IG introduced here exhibits high mechanical strength and high ionic conductivity. Its synthesis is simple and straightforward and is based on confining the IL 1-butyl-3-methylimidazolium bis(trifluoromethane sulfonyl)imide, [Bmim][NTf_2_] within a new organic–inorganic hybrid host. The organic–inorganic network is obtained by polymerization/condensation of methyltrimethoxysilane (MTMS), tetraethoxysilane (TEOS), and methyl methacrylate (MMA), which provides good mechanical strength and rapid shape recovery characteristics to the final IG. A silane coupling agent KH-570 (3-methacryloxypropyltrimethoxysilane) is used to tightly connect the inorganic and the polymeric components (SiO_2_ and PMMA, respectively) providing the intimate contact between the components and thus excellent mechanical properties to the matrix. IL addition leads to robust but flexible IGs, which can be deformed by an external force and quickly return to the original shape after the external force is removed. The IG exhibits a high ionic conductivity over a range of temperatures and thus is envisioned as a promising solid electrolyte in electronic devices. Finally, luminescent organisilica-containing carbon dots (CDs) can be homogeneously distributed in the IG to yield transparent, flexible, ion conducting, and blue light emitting monolithic materials.

## 2. Materials and Methods

Materials. MMA (99%), TEOS (99%), MTMS (99%), citric acid (99%+), KH-570 (3-methacryloxypropyltrimethoxysilane, 98%), and 3-(2-Aminoethylamino) propyl) trimethoxysilane (AEAPTMS, 99%) were obtained from Adamas Co., Ltd. (Shanghai, China). Azobisisobutyronitrile (AIBN) was procured from Energy Chemical Co., Shangai, China. [Bmim][NTf_2_] was purchased from Lanzhou Yulu Fine Chemical Co., Ltd., Lanzhou, China. Glucose was supplied by Xilong Scientific Co., Ltd., Shangtou, China. EtOH (analytical purity) was obtained from Sinpharm chemical reagent Co., Ltd., Ningbo, China. All chemicals were used as received.

CD synthesis. Citric acid and AEAPTMS, (mass ratio = 1:20) were mixed in a Teflon reactor under stirring at room temperature for 0.5 h. The resulting solution was heated to 180 °C for 12 h to obtain a yellow uniform liquid. The liquid was extracted with n-hexane to remove unreacted substances. Finally, the residual liquid was removed by rotary evaporation at 60 °C to obtain a gelatinous yellow powder.

IG synthesis. A certain amount (2 mg) of CDs were mixed with TEOS (2.08 g, 0.01 mol) and MTMS (1.36 g, 0.01 mol) in a mixed solvent of ethanol and water (ethanol/water ratio = 1) under stirring for 10 h at room temperature to obtain a uniform liquid. Then, MMA (4 g, 0.04 mol) and KH-570 (0.62 g, 0.0025 mol) were added to the above solution, and the temperature was increased to 65 °C for 40 min using an oil bath. After that, 8 mg AIBN and [Bmim][NTf_2_] (0.5 g) were added to the solution, and the reaction mixture was kept at 60 °C for 24 h. Once the gel solidified, the organic–inorganic luminescent IGs were collected. 

Characterization. X-ray diffraction was done on a Rigaku Ultima IV (Rigaku, Japan) with a scanning angle range (2θ) from 7° to 85°, with a scan speed of 5°/min. Attenuated total reflection infrared spectroscopy was done on a ThermoFisher iS50 (Thermo Fisher Scientific, Waltham, MA, USA) from 500 to 4000 cm^−1^. TGA and differential scanning calorimetry experiments were done on a TA Instruments SDT Q600 TGA (TA Instruments, New Castle, Delaware, USA) from room temperature to 800 °C in air with a heating rate of 10 °C min^−1^. A steady-state transient fluorescence spectrometer (FLS-920, Edinburgh, UK) was used for analyzing the fluorescence absorption and emission phenomena of IGs. Ionic conductivities were measured using a Solartron SI-1260 (Solartron, Zurich, Switzerland) between 25 and 100 °C and frequencies from 10 to 10^6^ Hz. Field-emission transmission electron microscopy images were obtained on a Talos F200S TEM/STEM (Thermo Fisher Scientific, Waltham, MA, USA) with a S-FEG Schottky gun operated at 200 kV. The sample was dispersed with ethanol under sonication for 1 min and then dipped on a Cu grid for TEM operation.

## 3. Results

**Figure 1:**
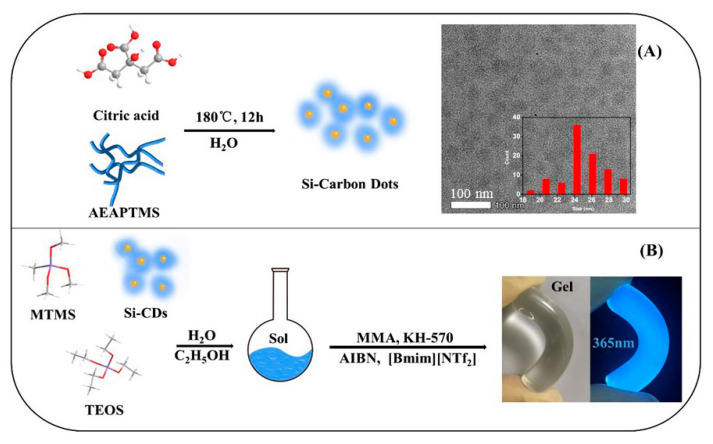
Preparation of carbon dots (CD) (**A**) and an organic–inorganic hybrid ionogel (IG) (**B**). AEAPTMS: (3-aminopropyl)triethoxysilane; MTMS: methyltrimethoxysilane; TEOS: tetraethoxysilane; MMA: methylmethacrylate; AIBN: azobisisobutyronitrile; KH-570: 3-methacryloxypropyltrimethoxysilane.

Figure 1 shows that a highly flexible, transparent, and luminescent IG can be fabricated via a two-step process. First, high-brightness blue fluorescent organosilica-modified CDs were synthesized by a simple one-pot hydrothermal method from citric acid and (3-aminopropyl)triethoxysilane (AEAPTMS) as raw materials. The particle sizes of the resulting CDs were roughly distributed between 20 and 30 nm (see the transmission electron micrograph in Figure 1A).

TEOS hydrolyzes to form a SiO_2_ network, which has a relatively poor mechanical stability due to the brittleness of the SiO_2_. To obtain a material with good mechanical properties, TEOS and MTMS were chosen as inorganic precursors [41], and MMA was selected as an organic component. The bifunctional coupling agent KH-570 was a key component in the material, because it directly bonded the organic component PMMA to the inorganic component SiO_2_. During the sol-gel process, silane hydrolysis and subsequent silanol condensation formed Si-O-Si bonds (i.e., silica), as by-products water and ethanol formed. At the same time, the free radical copolymerization of MMA and KH-570 was initiated by the thermal decomposition of AIBN, resulting in the formation of PMMA. PMMA provided additional flexibility and stability to the silica network.

Table 1 summarizes the influence of the reactant ratio on the IG properties, such as quantum yield and mechanical properties. Clearly, the quantum yield of the IG increased to 68–69% after the IL addition. With the addition of organic components, the mechanical properties of the IG also improved. However, the addition of large amounts of the IL led to less favorable mechanical properties.

**Figure 2:**
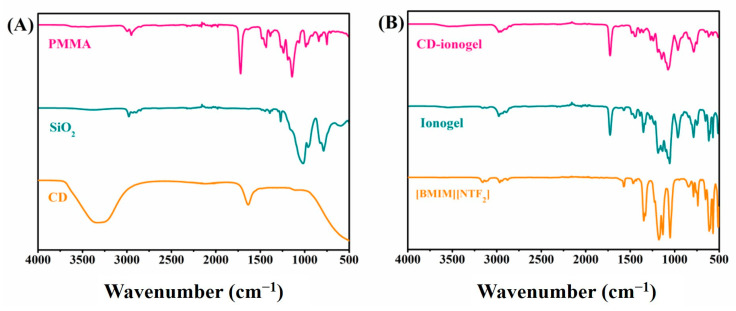
(**A**) Attenuated total reflection infrared (ATR-IR) spectra of PMMA, SiO_2_, and CDs. (**B**) ATR-IR spectra of [Bmim][NTf_2_], the ionogel with a TEOS/MTMS/MMA feed mass ratio of 1:1:4 and 0.5 g IL, and the CD–ionogel A5.

The attenuated total reflection infrared (ATR-IR) spectra of the Si–CD mixture were measured after the dialysis of the CD dispersion against water (Figure 2A). The spectra show bands at 1635 and 3330 cm^−1^ that can be assigned to the stretching vibration of C=O and –OH, respectively. This indicated that the Si-CD mixture contained abundant carbonyl and hydroxyl functional groups. Bands at 1019 cm^−1^ and 960 cm^−1^ stemmed from Si–O vibrations, and the –CH_3_ stretching vibration appeared at 2980 cm^−1^. The spectra of pure PMMA show a strong band at 1724 cm^−1^ from the PMMA carbonyl group. The bands at 2951 cm^−1^, 1432 cm^−1^, 1228 cm^−1^, 842 cm^−1^, and 743 cm^−1^ originated from aliphatic C–H vibrations. The bands at 1271 cm^−1^, 1138 cm^−1^, and 985 cm^−1^ were assigned to C–O–C stretching vibrations of PMMA. The bands at 3162 cm^−1^ and 3123 cm^−1^ were assigned to the C–H vibration of the [Bmim]^+^ cation. The band at 1566 cm^−1^ stemmed from C–C and C–N bending vibrations in [Bmim][NTf_2_]. The IR spectra of the IG show bands of both PMMA and IL, indicating successful IG formation.

The IR spectrum of [Bmim][NTf_2_] (Figure 2B) shows bands at 3264 cm^−1^, 1572 cm^−1^, 1179 cm^−1^, and 612 cm^−1^ that can be assigned to the vibration of C–H on the imidazolium ring [Bmim]^+^. Bands at 2970 cm^−1^, 847 cm^−1^, and 737 cm^−1^ were assigned to −CH_2_- and −CH_3_ vibrations on the alkyl side chains. Bands at 1344 cm^−1^ and 1050 cm^−1^ stemmed from vibrations of the sulfonyl group in the anion [NTf_2_]^−^. The IR spectra of the IG show the combined characteristic peaks of Si–CD, [Bmim][NTf_2_], the organic component (PMMA), and the inorganic component (SiO_2_), indicative of homogeneous distribution of all constituents. 

**Figure 3:**
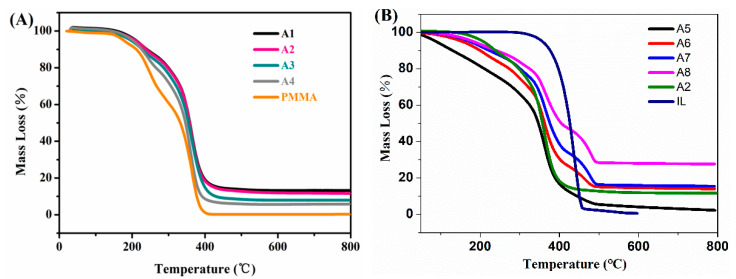
(**A**) TGA curves of materials A1–A4 with MMA/TEOS/MTMS monomer ratios of 1:1:2, 1:1:4, 1:1:6, and 1:1:8 along with data of PMMA. (**B**) TGA curves of IGs A5–A8 with 0.5, 1, 1.5, and 2 g of [Bmim][NTf_2_].

The TGA data of the organic–inorganic hybrid matrices and the IGs are shown in Figure 3. The first significant weight loss onset temperature of PMMA was below 200 °C. Between room temperature and 300 °C, PMMA lost about 40% of its mass; the mass loss between 300 °C and 400 °C was about 60%. At 400 °C, the PMMA was essentially decomposed, and no residual mass was left. As the curves are broad, detailed assignments were difficult, but the weight losses likely stemmed from overlapping signals from solvent evaporation as well as the evaporation and decomposition of the residual monomer and then, at higher temperatures, resulted from PMMA depolymerization and vaporization along with polymer degradation and carbonization.

Figure 3A also shows that as the fraction of the inorganic component increases, the thermal stability of the matrix gradually increased. In contrast to those of PMMA, the weight losses of the four organic–inorganic gels were 68%, 71%, 76%, and 79%, respectively, with a residue of silica. Likely, the decomposition processes are identical to those just described above. The thermal stability of the IGs was gradually improved upon IL addition. This indicated that the IL acts as a thermal stabilizer, similar to previous reports [11,28] The first weight loss in the TGA data between room temperature and 200 °C is likely again due to the loss of the organic solvent, the moisture, and the residual monomer. Between 200 °C and 400 °C, a rather distinct weight loss was visible. Presumably, it stemmed from the decomposition of the organic components, including the IL. Indeed, as the ILs content increased, the thermal stability of the IG increased. The relative intensities of these two weight losses were proportional to the weight fractions of the polymer and IL in the IG, respectively. The TGA, thus, showed that the IL significantly increased the thermal stability of the IG. However, the ratios of the precursor for A2 and A5 were the same, but their thermal stability displayed different behaviors. This can be explained by the fact that the polymerization reaction during the formation of the organic-–inorganic hybrid was affected by the ILs. We speculate that the IL could reduce the polymerization degree of the PMMA–SiO_2_ matrix, although this would contradict common knowledge of radical polymerization in ILs.

**Figure 4:**
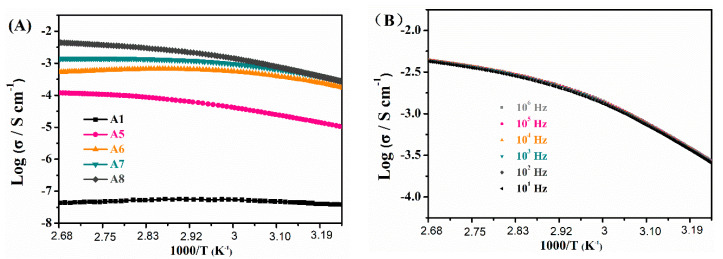
(**A**) Ionic conductivities of IGs A5 to A8 vs. temperature. The sample A1 (matrix only, no IL) is shown for comparison. (**B**) Ionic conductivities of A8 at different frequencies and temperatures.

The ionic conductivities (*σ*) of the matrix and the IGs between 30 °C and 100 °C at different IL weight fractions were also investigated. The neat polymer exhibited a very low *σ* of about 3.3 × 10^−8^ S cm^−1^. After the loading of low IL concentrations, the σ values of the IGs were, not surprisingly, much higher than that of the neat polymer. Furthermore, *σ* increased with increasing IL content by several orders of magnitude (the σ values of A5–A8 are 1.2 × 10^−4^, 5.5 × 10^−4^, 1.4 × 10^−3^, and 4.3 × 10^−3^ S cm^−1^, respectively, at 100 °C). Figure 4B shows an example of *σ* vs. frequency. The data clearly show that the relationship between *σ* and frequency did not change; this indicated that the IGs were stable and that the materials only showed a very slight dependence of the conductivity in frequency sweeps. The Arrhenius plots of the temperature dependency of σ exhibit complex upward/downward curved profiles, which can be fitted with the Vogel-Tamman-Fulcher (VTF) equation for the conductivities of electrolytic materials. The VTF equation was written as:

*σ = σ*_0_ exp[−*B*/(*T* − *T*_0_)],

where the constants, *σ*_0_ (S cm^−1^), *B* (K), and *T*_0_ (K) are adjustable parameters.

For application of IGs in the nascent field of flexible matter such as a soft robotics, good mechanical properties under strong stress are required. The mechanical analysis of the IGs showed that they can reversibly be compressed without permanent deformation or breakage. This indicated that the IG combined the hardness of the inorganic component with the flexibility of the organic component, and the gel material could quickly recover its shape after removing the applied external force. 

**Figure 5:**
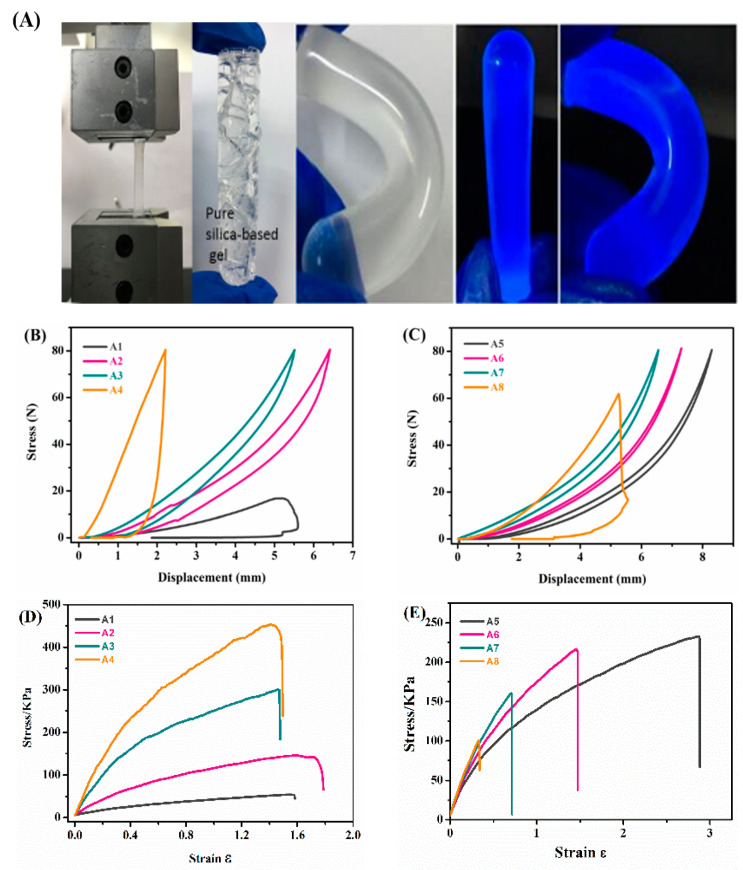
(**A**) Photographs of an IG in the mechanical analysis setup, a pure silica gel after mechanical treatment, a bent hybrid ionogel, and two images of an ionogel under UV irradiation. (**B**) Compressive stress–strain curves of A1, A2, A3, and A4. (**C**) Compressive stress–strain curves of A5, A6, A7, and A8. (**D**) Tensile stress–strain curves of A1, A2, A3, and A4. (**E**) Tensile stress–strain curves of A5, A6, A7, and A8.

Prior to analysis, all samples were fabricated in uniform shape with a cylindrical sample of 1 cm in diameter and height. Figure 5 shows that all materials exhibited a certain degree of plasticity within an effective elastic deformation range. By exerting and releasing a certain force until 80 N, a hysteresis loop was observed. Figure 5B,C clearly shows that there was a direct dependence of the mechanical properties from the organic and inorganic fractions in the materials. When the inorganic content increased, the material exhibited a higher brittleness and lower elasticity. For example, sample A1 broke at only 20 N, and the compression strain only reached 0.515 while the tensile strain reached 1.582. The IGs with higher organic content showed a higher compression strain of 0.619 (sample A2) and a higher tensile strain of 1.788 (sample A2). This indicated that the idea of an organic–inorganic hybrid is a powerful strategy to improve the mechanical properties of the gels.

In terms of the role the IL plays on the mechanical behavior, the true IGs (A5 to A8) with the ILs fractions of 30–69 wt % endowed an additional flexibility of the materials. Specifically, Figure 5C,E shows that the compressive plastic strain was increased from 0.619 (A2) to 0.840 (A6) and the tensile plastic strain was increased from 1.788 (A2) to 2.848 (A5). Optically, for the IG with an initial length of 19.5 mm, the maximum deformation displacement reached 56.1 mm. However, with the further increase of the IL content, the gel material became relatively soft, leading to low hardness, and exhibited poor mechanical properties and poor plasticity. Overall, by the proper selection of organic components or by IL incorporation, the mechanical properties of IGs can be tuned in a relatively wide range, producing materials that may be suitable in soft electronic device applications.

**Figure 6:**
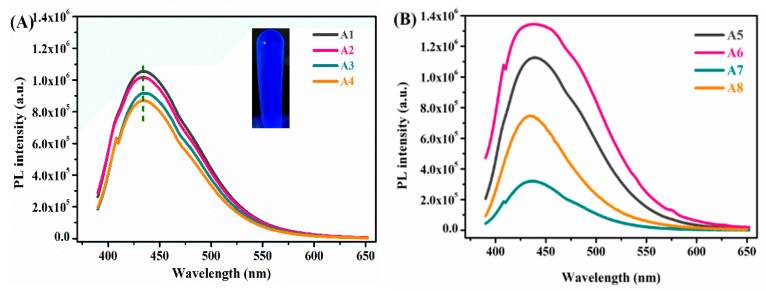
Fluorescence spectra of luminescent gels with different component ratios under 365 nm UV irradiation. (**A**) Sample A1–A4; (**B**) Sample A5–A8.

Furthermore, to explore the application potential of the IGs, the as-prepared IGs were used as a support to disperse carbon nanoparticles. Here, luminescent CDs were homogeneously dispersed in the IGs-yielding transparent, flexible, ion conducting, and blue light-emitting monolithic materials. Figure 6 (inset) shows that all CDs-doped IGs emitted a bright blue fluorescence under 365 nm UV irradiation. The further investigation of the absorption and emission behavior showed that the strongest emission was observed upon irradiation at 360 nm with an emission maximum at ca. 430 nm and a shoulder at 460 nm, which were mainly attributed to the emissions of CDs. Interestingly, when the IL was added, the fluorescence quantum yields of the gel materials increased significantly from 29% to 69%. Presumably, the presence of the IL provided a more homogeneous distribution and a more stable dispersion of the CD in the PMMA–SiO_2_ polymer. This results in the less aggregation of the CDs favoring an improved intensity of luminescence. The quantum yield (QY) was not linearly related to the IL contents. Rather, the QYs of sample A5 and A6 were 67% and 69%, respectively. In general, the addition of IL improved the quantum yield when compared with the IL-free materials, even for A7 (45%) and A8 (48%), which contained relatively little IL. This indicated that lower IL contents facilitate the dispersion and stability of CDs and possibly also led to an improved stability of excited states while excessive IL fractions such as in A7 and A8 could result in luminescence quenching.

**Figure 7:**
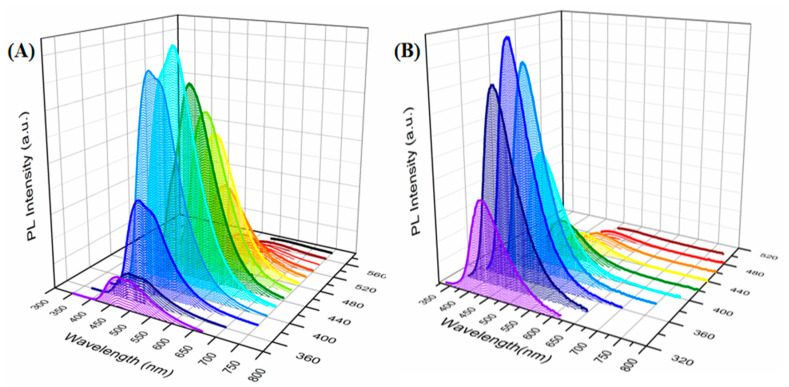
(**A**) Photoluminescence spectra of the Si–CD aqueous dispersion with excitation wavelengths from 340 nm to 580 nm at 20 nm increments. (**B**) Photoluminescence spectra of IG A5 with excitation wavelengths from 320 nm to 500 nm at 20 nm increments.

Excitation-dependent emission is a well-known property of CDs, which could be also observed in the current materials. As shown in Figure 7A, the emission spectra of the CDs in aqueous solutions were observed from 473 nm (blue emission) to 635 nm (red emission) upon different wavelength excitations, in which the surface PL emission energy level may come from the distribution of different emission trap sites on the surface of each CD. Different from those in aqueous solutions, the red-shift phenomena could be observed in the IG, accompanied with narrower peaks and increasing intensities. Specifically, the relatively strong emissions in the gels ranged from 439 nm to 551 nm, which may be attributed to the lower CD concentration in the IG providing less reabsorption of the excitation light by the remaining CDs. However, for high concentrations of CDs, the broad emission peaks may result from the continuous reabsorptions.

## 4. Conclusions

A novel organic–inorganic hybrid matrix for advanced IGs has been reported. The major advance over existing IGs is the fact that the combination of sol-gel chemistry with radical polymerization leads to robust, yet flexible, IGs with adjustable mechanical properties, depending on the precursor ratios. Moreover, the resulting materials were multifunctional soft hybrids combining advantageous mechanical properties with interesting optical and electrical properties. Of particular interest, the silane coupling agent 3-methacryloxypropyltrimethoxysilane acted as a bifunctional crosslinker tightly connecting the polymer network with the silica network, providing access to homogeneous materials with tunable properties. Overall, IGs with higher organic content show higher compression strain and tensile strain. The IL [Bmim][NTf_2_] immobilized within the flexible gel provided the material with an ionic conductivity as high as ca. 0.04 S cm^−1^ at 50 °C. This indicated that the idea of an organic–inorganic hybrid is a powerful strategy to improve the mechanical and electrical properties of soft matter. Overall, the materials introduced here are prototypes for the development of the next generation of multifunctional IGs with broad application potential.

## Figures and Tables

**Table 1:** Feed mass ratios, ionic liquids (IL) contents, CD contents, quantum yields (QYs), and plastic strains of the IGs.

Samples	TEOS/MTMS/MMA Feed Mass Ratio	IL (g)	CD (mg)	QY (%)	Tensile strain	Compressive Strain	TG Decomposition (°C)
A1	1:1:2	0	2	36%	1.582	0.515	177.2
A2	1:1:4	0	2	32%	1.788	0.619	177.5
A3	1:1:6	0	2	30%	1.476	0.557	177.5
A4	1:1:8	0	2	29%	1.496	0.213	178
A5	1:1:4	0.5	2	67%	2.884	0.698	99
A6	1:1:4	1.0	2	69%	1.474	0.840	162
A7	1:1:4	1.5	2	45%	0.715	0.747	189
A8	1:1:4	2.0	2	48%	0.340	0.544	192
